# Sir Benjamin Brodie, Bart

**Published:** 1863-01

**Authors:** 


### SIR BENJAMIN BRODIE, BART.

Full of professional honours and well-stricken in years, this eminent surgeon and excellent man rests from his labours. Few have attained to such high distinction. Few have so well merited the position which he so long occupied. The unanimous accord of the medical profession and of the public had for many years placed him above rivalry. Successive sovereigns had conferred upon him the highest dignity awarded to the members of his profession—that of personal attendance on Royalty. His individual amiability confirmed what his genius accomplished. Our pride in him left no room for jealousy of his success. To trace the progress of Sir Benjamin Brodie would be to review the advance of scientific surgery for the last half-century. An experimental philosopher, who regarded the organism as materials for the exercise of life. An analytical inquirer, who traced the operations of life through the molecular constitution of matter. A practical anatomist, familiar with the intricate machinery of the body. A profound physiologist, who interpreted the book of Nature from the writing of her works. An operating surgeon, whose manual dexterity placed the mechanism of his art first amongst the sciences. A practical physician, whose treatment of disease reduced the philosophy of medicine to the noblest of arts. A great man, gifted with an order of intellect, which, in any age, would have accomplished mighty results, and which, in a period remarkable for the boldness and grandeur of its progress, worthily achieved for him a first position amongst the master-minds of the day. Such was Sir Benjamin Brodie. It is not for us to do more than honour his memory and emulate his example. It is ordained for each once to die. His was a placid termination to an honourable and a blameless life. In common with all we express our affectionate respect for the illustrious dead, and indulge our selfish sorrow at his departure from amongst us.

This is not the occasion to review Sir Benjamin Brodie's contributions to that special department of medical science to which this Journal has been more particularly directed. It is enough to say that all that he has written he has written well. While the sense of our professional bereavement is yet keen, we forbear from any more lengthened investigation of that brilliant career which our cotemporaries have so well placed in detail before the profession. Sir Benjamin Brodie yet lives—lives in the present, and lives for time. His eulogy will ever be perpetuated where suffering succumbs to the treatment his profound perception bequeathed to those who follow him. His epitaph will not be erased from the hearts of any whose privilege it has been to know this great and good man.

